# The Stapled Peptide PM2 Stabilizes p53 Levels and Radiosensitizes Wild-Type p53 Cancer Cells

**DOI:** 10.3389/fonc.2019.00923

**Published:** 2019-09-19

**Authors:** Anja Charlotte Lundgren Mortensen, Diana Spiegelberg, Christopher John Brown, David Philip Lane, Marika Nestor

**Affiliations:** ^1^Department of Immunology, Genetics and Pathology, Uppsala University, Uppsala, Sweden; ^2^Department of Surgical Sciences, Uppsala University, Uppsala, Sweden; ^3^Agency for Science, Technology and Research (A*STAR), Singapore, Singapore; ^4^Science for Life Laboratory, Department of Microbiology, Tumor and Cell Biology, Karolinska Institutet, Stockholm, Sweden

**Keywords:** p53, wild-type p53, radiosensitization, external beam radiation therapy (EBRT), PM2, spheroid apoptosis

## Abstract

The tumor suppressor p53 is a key mediator of cellular stress and DNA damage response cascades and is activated after exposure to ionizing radiation. Amplifying wild-type p53 expression by targeting negative regulators such as MDM2 in combination with external beam radiotherapy (EBRT) may result in increased therapeutic effects. The novel stapled peptide PM2 prevents MDM2 from suppressing wild-type p53, and is thus a promising agent for therapeutic combination with EBRT. Effects of PM2 and potential PM2-induced radiosensitivity were assessed in a panel of cancer cell lines using 2D cell viability assays. Western Blot and flow cytometric analyses were used to investigate the mechanisms behind the observed effects in samples treated with PM2 and EBRT. Finally, PM2-treatment combined with EBRT was evaluated in an *in vitro* 3D spheroid model. PM2-therapy decreased cell viability in wild-type p53, HPV-negative cell lines. Western Blotting and flow cytometry confirmed upregulation of p53, as well as initiation of p53-mediated apoptosis measured by increased cleaved caspase-3 and Noxa activity. Furthermore, 3D *in vitro* tumor spheroid experiments confirmed the superior effects of the combination, as the only treatment regime resulting in growth inhibition and complete spheroid disintegration. We conclude that PM2 induces antitumorigenic effects in wt p53 HPV-negative cancer cells and potentiates the effects of EBRT, ultimately resulting in tumor eradication in a 3D spheroid model. This strategy shows great potential as a new wt p53 specific tumor-targeting compound, and the combination of PM2 and EBRT could be a promising strategy to increase therapeutic effects and decrease adverse effects from radiotherapy.

## Introduction

Today up to 50% of all cancer patients receive at least one dose of external beam radiotherapy (EBRT) (1–4). With continuous technological increases over the last few decades, it has evolved into one of the most well-established and successful non-invasive cancer therapy regimens (5, 6). Improvements to EBRTs such as intensity modulated radiotherapy (IMRT) and volumetric modulated arc therapy (VMAT) are undergoing continuous development, exemplifying that EBRT as a field still has room for improvement (5, 7–9). One such improvement is the use of radiosensitizers, where sensitizing tumor cells to radiation damage may further increase the radiotherapy success rate. Tumor cell radiosensitization could also allow reductions in radiation dose, thus lowering the risk of adverse effects (10–12).

The transcription factor p53 is a promising target for radiosensitization, as it plays a key role in both DNA repair and radiation response mechanisms (13). p53 is upregulated after exposure to ionizing radiation and drives a variety of cellular responses in damaged cells, where the specific response depends quantitatively on the level of p53 accumulation (13–15). The outcome of p53 accumulation is complex and often cell type specific (16). While low levels of wt p53 leads to cell cycle arrest, higher levels may result in apoptosis, senescence or autophagy. In the event of apoptosis, it is linked to transcription of pro-apoptotic proteins such as Phorbol-12-myristate-13-acetate-induced protein 1 (Noxa) (14, 17, 18).

Due to its key role, p53 is commonly inactivated in cancer cells to avoid the induction of apoptosis, either through mutations in p53, or by accumulating mutations in p53 regulatory factors while still maintaining wild-type p53 (wt p53). While wt p53 expression remains intact in around 50% of all cancers, it is often suppressed by dysfunctional activation pathways, for example epigenetic silencing of p14 ARF expression or overexpression of negative feedback regulators such as murine double-minute 2 (MDM2) and its structural homolog, murine double-minute X (MDMX) (19, 20). MDM2/X are p53 target proteins that bind and block the transcriptional activity in an autoregulatory feedback loop and mark the p53 protein for ubiquitination (21).

Previous studies have demonstrated that wt p53 expression can be amplified by targeting MDM2/X, which has been shown to result in tumor regression *in vivo* (22). Inhibiting the MDM2-p53 protein-protein interaction causes wt p53 accumulation in the cancer cells, which may eventually lead to cell cycle arrest or cell death. Promising pre-clinical data has led to several MDM2/X-p53 inhibitors currently undergoing clinical trials (23, 24). However, none of the current clinical trials are exploring combined EBRT and MDM2/X-p53 inhibition therapy, which could PM2 therapy potentially provide further utility within the growing field of MDM2-p53 inhibitors.

The present study involves PM2, which is a novel stapled peptide targeting the MDM2/X-p53 interaction (25). Like most MDM2/X-p53 inhibitors, PM2 mimics the amino acid sequence of wt p53 that is bound by MDM2/X (26, 27). “Stapling” in this context means that a covalent hydrocarbon linker has been introduced between two non-adjacent amino acids, thus connecting turns of the peptide's α helix resulting in greater stability (21, 26, 27). The stabilization of the peptide's secondary structure, in addition to increasing its affinity for MDM2/X by reducing the entropic cost of binding, also results in an increase in its *in vivo* half-life. The use of staple peptides, which have a much more comprehensive network of interactions with MDM2 than small molecule inhibitors such as Nutlin-3, have been shown to bind to and antagonize Nutlin-3-resistant MDM2 (26, 27).

In a recent study we have established the *in vivo* potential of PM2 as a radiotherapy potentiator in a wt p53 colorectal cancer model (28). In mice carrying wt p53 tumors, PM2 combined with radiotherapy prolonged median survival by 50%, whereas effects on p53^−/−^ tumors were negligible. This proof-of-concept study demonstrates the promise of this application *in vivo*, and suggests that a future clinical application of PM2 with radiotherapy in wt p53 cancers might improve tumor control. However, to enable such a scenario it is vital to thoroughly assess the effects of PM2 and EBRT on a larger scale, to validate p53-dependent effects, and most importantly to unravel the mechanisms behind the effects and the cellular fates of treated cells. Consequently, the aim of this study was to assess the potential antitumorigenic effects of the combination of EBRT and PM2 therapy as well as to determine the mechanisms behind the observed effects.

## Materials and Methods

### Cell Lines

The human squamous cell carcinoma cell lines UM-SCC-74A and UM-SCC-74B were kindly provided by Professor TE Carey (University of Michigan, USA) and cultured in Dulbecco's Modified Eagle Medium (DMEM) with 10% fetal bovine serum, 1% L-glutamine, 1% antibiotics (100 IU penicillin and 100 μg/ml streptomycin) as well as 1% non-essential amino acids (all from Biochrom Kg, Berlin, Germany). UT-SCC-45 (kindly provided by Dr. R. Grenman, Turku University Central Hospital, Finland) was cultured in DMEM with the abovementioned additives. HCT116 and A431 were purchased from American Type Culture Collection (Manassas, VA, USA). HCT116 was cultured in McCoy's modified Eagle medium with 10% fetal bovine serum, 1% L-glutamine and 1% antibiotics (100 IU penicillin and 100 μg/ml streptomycin) and A431 was cultured in Ham's F10 with the same additives. H314 was purchased from the European Collection of Authenticated Cell Cultures (Salisbury, UK) in DMEM/Ham's F12 (1:1) with the abovementioned additives. Cells were incubated at 37°C with 5% CO_2_, and cultivated for no longer than 2 months at a time.

### PM2

The stapled MDM2-p53 antagonist, PM2 (MW = 1462.75Da), was produced at p53 Laboratory (A^*^STAR, Singapore) and dissolved in DMSO to a stock concentration of 10 mM and stored at −20°C.

### NanoBIT Mdm2:p53 PPI Cell Based Assay

HEK293 FT cells were seeded at a cell density of 5^*^10^5^ cells per well into a six-well plate and incubated overnight at 37°C and 5% CO_2_ in DMEM with 0.3 mg/ml glutamine, 100 IU/ml penicillin, 100 μg/ml streptomycin and 10% fetal calf serum. Each well was transfected with 2 μg of DNA consisting of a 1:1 ration of the sMBIT-Mdm2 fusion and LgBIT-p53 fusion vectors (PROMEGA). Each transfection was performed following the manufacturer's instructions with a 3:1 ratio of FuGene transfection reagent (PROMEGA) to DNA in 0% FCS Opti-MEM no red phenol media. Cells were incubated overnight and then washed with 1 ml of PBS, followed by trysinization and re-suspension in Opti-MEM media with 0% FCS. The cell suspension was then adjusted to 2.2 × 10^5^ cells per ml.

Re-suspended HEK-293 FT cells were centrifuged at 1,000 rpm for 5 min at room temperature. Ninety microliter of the cell re-suspension was added to the wells of a white opaque 96-well plate. Stapled peptides were then titrated onto the 96-well plate using a suitable 2-fold dilution series from a 10 × stock solution containing 10% *v*/*v* DMSO. Control wells were also treated with a 10% DMSO only stock solution to yield a final residual DMSO concentration of 1% *v*/*v*. The 96-well plates were incubated for 4 h at 37°C, with 5% CO_2_. Wells were treated with a solution of Nano-Glo live substrate, which was prepared as per the manufacturer's instructions (PROMEGA). The plates were shaken for 1 min at 22°C and luminescence assessed after an additional 50 min using an Envision Multi-Plate reader (Perkin-Elmer, Waltham, MA, USA). NanoBit titration data was used to determine IC_50_ values for drug potency by fitting 4-parameter logistic curves using GraphPad Prism 7.0.

### Radiation

Cells were irradiated using ^137^Cs gamma-ray photons at a dose-rate of ~1 Gy/min (Best Theratronics Gammacell® 40Exactor, Springfield, USA).

### Cell Viability Assays, 2,3-Bis(2-Methoxy-4-Nitro-5-Sulfophenyl)-2H-Tetrazolium-5-Carboxanilide Salt (XTT)

Five-hundred to ten thousands cells were seeded in flat-bottomed 96-well plates and incubated for 48 h prior to irradiation with 2Gy. Three hours post-irradiation, PM2 was added in concentrations spanning 0–40 μM. Cells were incubated at 37° for 4–5 days until 80–90% confluence had been obtained. A mix of XTT Activation Reagent and XTT Reagent was added according to the American Type Culture Collection 30–1011 K protocol (Manassas, VA, USA). Plates were incubated for up to 6 h at 37° and absorbance was measured by using a BioMark Microplate Reader (Bio-Rad Laboratories AB, Solna, Sweden).

### Western Blot

Cells were incubated for at least 24 h prior to irradiation (2 Gy) and/or PM2 treatment, where PM2 (20 μM) was added 3 h after irradiation. Whole-cell lysates were prepared according to standard protocols. Protein concentrations were measured using a DeNovix DS-11 spectrophotometer (DeNovix Inc., Wilmington, DE, USA). Samples were separated on 4–12% Bis-Tris SDS gels and transferred to a nitrocellulose membrane (ThermoFisher Scientific, Uppsala, Sweden) by wet-transfer blotting. The membrane was blocked for 1 h in PBS with 5% BSA before incubation with primary antibodies targeting p53, caspase-3, NaK-ATPase and GLB1 (DO-1 (ab1101), ab13847, ab76020 and ab128993, AbCam, Cambridge, UK) and β-actin (A5441, Sigma Aldrich Sweden, Stockholm, Sweden) at 4°C overnight. After washing with PBS with 1% Tween-20, the membrane was incubated with respective and species-specific Horse Radish Peroxidase-labeled secondary antibody (ThermoFisher Scientific, Waltham, MA, USA) and stained with electro-chemiluminescent solution (Immobilon, Millipore, Bedford, USA). Immunoreactive bands were visualized with a CCD camera (SuperCCD HR, Fujifilm, Japan) and analyzed in ImageJ version 1.48 (NIH, Bethesda, MD, USA).

### Flow Cytometry

Cells were incubated for at least 24 h prior to irradiation and/or PM2 treatment. Twenty micrometer PM2 was added with a 3-h delay after irradiation. Cells were collected and fixed in 70% ethanol at 6, 12, 24, 48, 72, and 96 h post-irradiation and stored at −20°C. For cell cycle analyses, 6, 12, 24, and 48 h samples were rehydrated and washed twice with PBS and stained with DAPI (Sigma Aldrich Sweden, Stockholm, Sweden) using 1 μg/ml for 30 min. 24, 48, 72, and 96 h samples were stained with caspase-3 and Noxa (ab13847 (1:500) and ab13654 (1:1,000), AbCam, Cambridge, UK) overnight at 4°C, followed by incubation with fluorescent labeled secondary antibodies for 90 min (ab 1:400) at room temperature. Analyses were performed using a BD LSR Fortessa flow cytometer (Becton Dickinson Biosciences, San Jose, USA). Data analyses for cleaved caspase-3 and Noxa were performed with BD FACSDiVa (Becton Dickinson Biosciences, San Jose, USA), while cell cycle analyses of exclusively viable cells were performed using FlowJo (Becton, Dickinson Biosciences, San Jose, USA). Coefficient of variation (CV) values, were below seven for all samples.

### 3D Assays

For liquid overlay 3D spheroid formation, 96-well plates were coated with 0.15% agarose dissolved in PBS with 1% penicillin/streptomycin. 1000 UM-SCC-74B cells/well were seeded and incubated at 37°C for 3 days prior to treatment. The standard dose of 20 μM of PM2 was added 3 h after irradiation. Half of the medium was replaced every 48 h for the first 10 days, thereafter every 4 days. Samples with repeated PM2 treatments received a new 20 μM dose despite removing of half of the incubation medium. Images were obtained every 2–4 days using a Canon EOS 700D camera mounted on an inverted Nikon Diaphot-TMD microscope. The images were analyzed using ImageJ version 1.48 (NIH, Bethesda, MD, USA), by measuring the surface area of each spheroid and calculating the volume, assuming each spheroid retained a spherical form. Each spheroid was normalized to its own starting volume at the start of treatment (Day 0, growth ratio = 1). Spheroids exceeding a volume of 600 μm^3^ were excluded (i.e., terminated) from further analyses as 600 μm^3^ is the maximum size possible to obtain in these settings with uncompromised growth conditions.

### Statistical Analyses

GraphPad Prism version 6.07 (GraphPad Software, San Diego, USA) was used for data processing and analysis. *p*-values were determined using unpaired student's *t*-test for comparison between two groups or one-way ANOVA followed by Tukey's multiple comparisons test, with *p* < 0.05 (^*^), *p* < 0.01 (^**^), *p* < 0.001 (^***^), and *p* < 0.0001 (^****^). For XTT assays cell viability was normalized for irradiated and unirradiated samples separately. Thus, an observed significant difference in viability between combination treated samples and solely PM2-treated samples, was considered as the result of PM2 potentiating the effects of radiation. A modified approach to the coefficient of drug interaction (CDI) was determined as: CDI = AB/(A^*^B), where AB was the ratio of the combination treatment to controls and A or B was the ratio of radiation or PM2 treatment to controls. CDI ≤ 0.7 equaled significant synergistic effect, CDI ≤ 1 equaled additive effect and CDI > 1 equaled antagonistic effect (29).

## Results

### PM2 Treatment Decreases Cell Viability and Radiosensitizes wt p53 Cells in Monolayer Cultures

**Figure 1:**
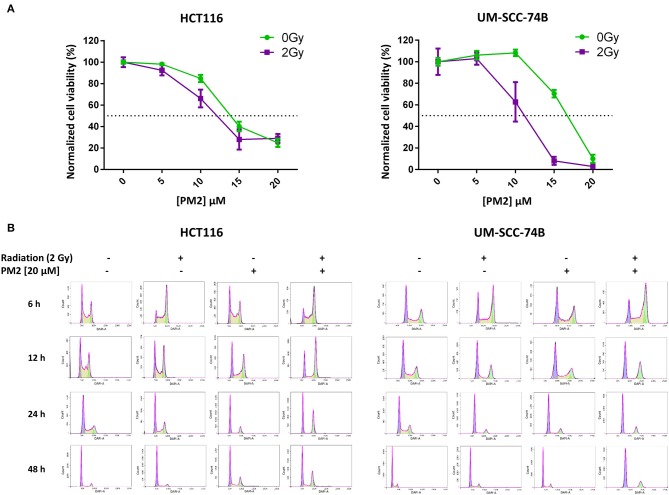
**(A)** Cell viability (XTT) response to treatment with 0–20 μM of PM2 of UM-SCC-74B and HCT116 without external radiation (green) and in combination with 2 Gy of EBRT (purple). Error bars presented as 95% confidence intervals, *n* = 6. Please note that irradiated cells were normalized to survival at 2 Gy, 0 μM PM2, whereas un-irradiated cells were normalized to survival at 0 Gy, 0 μM PM2 in order to compensate for the effects of radiation. Dotted line indicated 50% viability of controls. **(B)** Cell cycle distribution of viable UM-SCC-74B and HCT116 cells at 6, 12, 24, and 48 h post-treatment with either 2 Gy, 20 μM of PM2, or the combination, fitted according to FlowJo cell cycle analysis software. CV-values for all cell cycle analyses were below 7.

Viability assays (XTT) of six cancer cell lines treated with PM2, either with or without the addition of 2 Gy of external radiation, were performed to measure the efficacy of PM2 alone as well as its potential radiosensitizing effects (Table 1). PM2 treatment decreased significantly the viability of all confirmed wt p53, HPV negative cell lines at low doses, starting at ~10 μM for both HCT116 and UM-SCC-74A and at a slightly higher dose for UM-SCC-74B (Figure 1A, green lines). Increasing concentrations of PM2 resulted in reduced viability. An inhibitory concentration of 50% decrease (IC_50_) was measured at 16 and 17 μM of PM2 for UM-SCC-74A and UM-SCC-74B, respectively and 14 μM for HCT116. At concentrations of 20 μM of PM2, the average viability was reduced by 73 and 90% for UM-SCC-74A and UM-SCC-74B, respectively, and 75% for HCT116, compared to untreated controls. Combination treatment of PM2 and EBRT exhibited a significant drop in viability compared to PM2-monotherapy outcomes at low doses of PM2, starting at 10 μM for UM-SCC-74B cells (Figure 1A). The IC_50_ of the combination treatment was detected at 10 μM for UM-SCC-74B and 12 μM for HCT116. These IC_50_-values correlate with the NanoBIT assays using PM2 (IC_50_ value of 14.8 ± 0.5 μM). The NanoBIT assay further verified the specificity of the antagonistic properties of PM2 to MDM2, with no effect obtained by the scrambled peptide PM2^SCRAM^ (Supplementary Figure 1A). PM2 did not affect the two confirmed mutant p53 cell lines, A431 and H314 (Table 1; Supplementary Figure 1B). However, the combination therapy significantly decreased the viability of A431. The HPV positive cell line, UT-SCC-45, was unresponsive to PM2 therapy regardless of the addition of EBRT (Table 1; Supplementary Figure 1B).

**Table 1:** Panel of screened cell lines using 0–20 μM of PM2 and 0 or 2 Gy of EBRT.

**Cell line**	**p53 status**	**HPV status**	**Cancer**	**PM2 effect**	**IC_**50**_**	**Significant effect of combination**
A431	Single base mutation (30)	Negative	Epidermal (vulva)	No	n/a	Yes
H314	Two mutations (31)	Negative	HNSCC (floor of mouth)	No	n/a	No
UT-SCC-45	Wild-type^*^ (32)	Positive	HNSCC (floor of mouth)	No	n/a	No
UM-SCC-74A	Wild-type (33)	Negative	HNSCC (tongue)	Yes	16 μM	Yes
UM-SCC-74B	Wild-type (33)	Negative	HNSCC (larynx)	Yes	17 μM	Yes
HCT116	Wild-type (34)	Negative	Adenocarcinoma (colorectal)	Yes	14 μM	Yes

* *Wild-type-status is only assessed for exon 4–8. n/a, not applicable*.

### PM2 and Radiation Treatment Results in G2/M Phase Shift and S-phase Depletion

To investigate underlying mechanisms of PM2 therapy associated cytotoxicity, cell cycle analysis of viable cells was performed on both UM-SCC-74B and HCT116. A distinct G2/M-shift was observed as early as 6 h post-irradiation with 2Gy for both HCT116 and UM-SCC-74B (Figure 1B). Single modality treatments with 20 μM of PM2 did not induce a G2/M-shift for either of the cell lines. More than twice as many cells comprised the G2/M-phase of the dual treated samples than untreated controls for both cell lines throughout the 48 h timeframe (Table 2). An S-phase reduction occurred as early as 12 h post-treatment for dual treated samples for UM-SCC-74B and HCT116, followed by S-phase depletion at 24 h post-treatment (Table 2).

**Table 2:** S-phase and G2/M-phase development 6–48 h post-treatment with 2 Gy, 20 μM of PM2, or the combination, of viable UM-SCC-74B and HCT116 cells.

**UM-SCC-74B: S-phase**					**UM-SCC-74B: G2/M-phase**				
**Time post-treatment**	**0 Gy**	**2 Gy**	**PM2**	**Combination**	**Time post-treatment**	**0 Gy**	**2 Gy**	**PM2**	**Combination**
6 h	34.7%	39.9%	41.6%	42.9%	6 h	23.6%	34.21%	19.7%	37.9%
12 h	37.9%	17.6%	18.3%	7.31%	12 h	19.5%	29.2%	18.3%	39.4%
24 h	33.4%	12.7%	12.9%	2.89%	24 h	16.8%	13.5%	16.7%	23.8%
48 h	20.4%	20.1%	17.4%	5.37%	48 h	11.9%	8.99%	10.8%	20.6%
**HCT116: S-phase**					**HCT116: G2/M-phase**				
**Time post-treatment**	**0 Gy**	**2 Gy**	**PM2**	**Combination**	**Time post-treatment**	**0 Gy**	**2 Gy**	**PM2**	**Combination**
6 h	55.1%	49.4%	55.8%	44.9%	6 h	19.5%	41%	19.4%	39.9%
12 h	50.7%	39.1%	35.1%	17.3%	12 h	18%	31.5%	30.1%	53.8%
24 h	33.7%	28%	10.7%	5.51%	24 h	14.3%	22.8%	21.1%	42.7%
48 h	16.5%	16.2%	23%	17.5%	48 h	10.8%	8.57%	20.6%	29.9%

*Coefficient of variation (CV) values for all cell cycle analyses were below 7*.

### PM2 Upregulates Cleaved Caspase-3, Noxa, wt p53, and GLB-1

**Figure 2:**
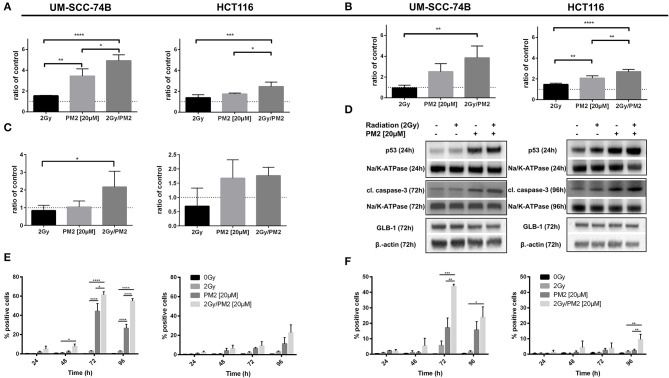
Western Blot analyses of p53-expression **(A)** at 24 h post-treatment of UM-SCC-74B and HCT116 samples with either 2 Gy, 20 μM of PM2, or the combination and cleaved caspase-3 expression **(B)** as well as GLB-1 expression **(C)** of UM-SCC-74B and HCT116 samples at 72 and 96 h post-treatment, respectively. NaK-ATPase was used as loading control for p53 and cleaved caspase-3, β-actin for GLB-1 expression. All samples were normalized to controls, represented as a dotted line at y = 1. *n* ≥ 3, error bars presented as SD. Representative Western Blot images **(D)** of p53, cleaved caspase-3 and GLB-1 expression of UM-SCC-74B at 24 h (p53) and 72 h (cleaved caspase-3, GLB-1) post-treatment, and HCT116 samples at 24 h (p53), 72 h (GLB-1), and 96 h (cleaved caspase-3) post-treatment. Flow cytometric analyses of cleaved caspase-3 **(E)** and Noxa-expression **(F)** of UM-SCC-74B and HCT116 samples at 24, 48, 72, and 96 h post-treatment with either 2 Gy, 20 μM of PM2, or the combination. *n* ≥ 3, error bars represent SD. Significance was determined using one-way ANOVA: *p* ≤ 0.05 (*), *p* ≤ 0.01 (**), *p* ≤ 0.001 (***), *p* ≤ 0.0001 (****).

The expression of wt p53, cleaved caspase-3 and GLB-1 following treatment with PM2 and/or radiation was visualized through Western Blotting. Western Blot analyses detected an increased expression of p53 in PM2-treated samples at 24 h post-treatment for both cell lines, with a significant increase between solely PM2-treated and combination treated samples (Figures 2A,D). Similarly, an increase in expression of the apoptotic marker cleaved caspase-3 was detected in all PM2-treated samples as well as irradiated samples, although the expression of the marker in the combination group was predominant (Figures 2B,D). Additionally, a significant increase of senescence marker, Galactosidase beta-1 (GLB-1), was detected at 72 h post-treatment in combination treated UM-SCC-74B samples compared to the irradiated samples. The same trend was observed in the HCT116 samples, where both the PM2-monotherapy and combination therapy resulted in a similar increase of GLB-1 compared to irradiated and control samples (Figures 2C,D). An increase in cleaved caspase-3 expression was confirmed through flow cytometry in PM2-treated samples at 48 h post-treatment, peaking at 72 and 96 h for UM-SCC-74B (40-fold increase compared to controls) and HCT116 (10-fold increase compared to controls), respectively (Figure 2E). The combination therapy samples had significantly higher expression levels of cleaved caspase-3 than PM2-treated samples for UM-SCC-74B (*p* ≤ 0.05). A similar trend was observed for HCT116. Expression levels of the p53-dependent pro-apoptotic protein Noxa correlated with the increase in cleaved caspase-3 levels. Near-3-fold and 4-fold increases in Noxa levels were observed after combination therapy compared to PM2 therapy alone for UM-SCC-74B and HCT116, respectively (*p* ≤ 0.01) (Figure 2F).

### Radiation Potentiates the Effects of PM2 in an *in vitro* 3D Tumor Model

**Figure 3:**
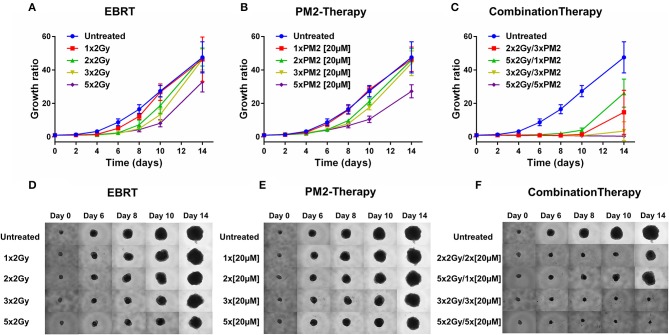
Therapeutic effect on growth of UM-SCC-74B spheroids of **(A)** EBRT, **(B)** PM2 therapy, and **(C)** combination therapy of EBRT and PM2 therapy (2 Gy and 20 μM of PM2 at 48 h intervals). Representative images of UM-SCC-74B spheroids during EBRT **(D)**, PM2 therapy **(E)**, and treatment combinations over time **(F)**. *n* ≥ 4, error bars represent 95% confidence intervals. Table 3 details the synergism between the treatments regimens.

**Figure 4:**
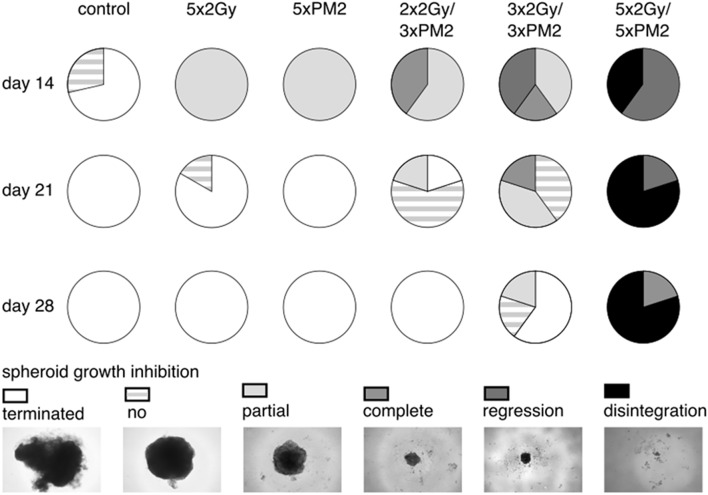
Spheroid response to selected repeated treatments of 20 μM of PM2, 2 Gy of EBRT, or combinations of the treatment modalities at day 14, 21, and 28 post-start of treatment, presented as parts of whole with representative images of the different therapeutic responses. Terminated, maximum assay dependent spheroid size reached (>600 μm^3^); no, no spheroid growth inhibition (spheroids with growth rates matching those of untreated controls); partial, partial growth inhibition (spheroids with reduced growth rates compared to untreated controls); complete, complete spheroid growth inhibition (spheroids with unchanged spheroid size post-start of treatment); regression, reclining spheroid size post-start of treatment; disintegration, dissolution of non-viable spheroids *n* ≥ 4.

In order to establish whether the potentiating effects of combining PM2 and radiation, as observed in monolayer cultures that were present in a 3D multicellular tumor spheroid system, the growth of wt p53 UM-SCC-74B spheroids was measured over time. EBRT and PM2 therapy had similar inhibitory effects on cell growth at repeated treatments (Figures 3A,B,D,E). Five repeated treatments of EBRT at 48 h intervals resulted in an average size of 69% of controls at day 14, whereas five repeated doses of 20 μM of PM2 resulted in an average size of 57%. Combining a single dose of PM2 with EBRT resulted in greater inhibitory effects than either treatment alone. The growth inhibiting effect of one PM2 treatment and radiation treatment was rather persistent, and started to fade after day ten (Figure 3C). Repeated doses of PM2 (2–5 × 20 μM) in combination with either a single radiation dose (2 Gy) or repeated doses (2–5 × 2 Gy) resulted in increased inhibitory effects in a repeated-dose-dependent manner (Figures 3C,F). By day 14, the majority of treatments had resumed normal or near-normal growth ratios, with the exception of repeated treatments of 2 × 2 Gy/3 × 20 μM, 3 × 2 Gy/3 × 20 μM, and 5 × 2 Gy/5 × 20 μM (Figure 3C). However, spheroids treated with three and five fractionated radiation doses in combination with a single dose of PM2 were reduced to a size of 75 and 55%, respectively, of untreated controls. Treatment with 5 × 2 Gy/5 × 20 μM of PM2 resulted in spheroid disintegration and cell death, which persisted beyond the end of the assay (Figures 3C,F, 4). Repeated treatments of 2 × 2 Gy/3 × 20 μM and 3 × 2 Gy/3 × 20 μM of PM2 also resulted in continuous growth inhibition throughout day 14; however, this combination did not result in spheroid disintegration (Table 3; Figure 4). All combination treatments demonstrated synergistic inhibitory effects on the proliferation rate of UM-SCC-74B spheroids prior to day 14 (Table 4).

**Table 3:** CDI-values of selected treatments of UM-SCC-74B spheroids on Day 10 and Day 14 post treatment.

**Day 10**					**Day 14**				
**Treatment**	**1 × 2 Gy**	**2 × 2 Gy**	**3 × 2 Gy**	**5 × 2 Gy**	**Treatment**	**1 × 2 Gy**	**2 × 2 Gy**	**3 × 2 Gy**	**5 × 2 Gy**
1 × 20 μM	0.705	0.507	0.493	0.472	1 × 20 μM	1.03	0.93	0.821	0.815
2 × 20 μM	n/a	0.209	n/a	n/a	2 × 20 μM	n/a	0.51	n/a	n/a
3 × 20 μM	0.412	0.122	0.244	n/a	3 × 20 μM	0.944	0.333	0.182	n/a
5 × 20 μM	0.398	n/a	n/a	0.201	5 × 20 μM	0.568	n/a	n/a	0.023

*CDI < 0.7 equaled a significant synergistic effect, CDI ≤ 1 equaled additive effect, and CDI > 1 equaled antagonistic effect. n/a, not assessed*.

**Table 4:** Median and maximum time (d) to spheroid termination due to excessive growth (volume >600 μm^3^) of selected treatments.

**Treatment**	**Control**	**2 × 2 Gy and 3 × PM2**	**3 × 2 Gy and 3 × PM2**	**5 × 2 Gy**	**5 × PM2**	**5 × 2 Gy and 1 × PM2**	**5 × 2 Gy and 5 × PM2**
Median time to termination (d)	14	21	28	21	21	21	Not reached
Maximum time to termination (d)	17	25	35	25	21	25	Not reached

*“Not reached” meaning no termination was present during the course of the assay*.

## Discussion

Several MDM2-p53 inhibitors are currently undergoing different stages of both preclinical and clinical evaluation. On the clinical level, the antagonists are primarily being tested either as single modality treatments or in combination with chemotherapeutic agents such as doxorubicin or cytarabine (23). Interestingly, even though wt p53 plays a central role in radiation response mechanisms, none of the current clinical trials are combining MDM2-p53 inhibitors with ionizing radiation. However, previous studies have demonstrated that stabilization of wt p53 may offer great potential to boost the therapeutic effects of radiotherapy (28, 35, 36). Consequently, the present study aimed to investigate whether the novel stapled MDM2/X-p53 inhibitor PM2 can potentiate the therapeutic effects of ionizing radiation in a panel of cell lines, the synergistic potential of various combinations of doses and fractionations, as well as the underlying mechanisms behind the effects.

In the present study, a single dose of PM2 (20 μM) impaired cell viability in all three wt p53 cell lines grown in monolayer. Furthermore, after a single dose of ionizing radiation (2 Gy), the effects of PM2 were significantly amplified (Table 1). The amplification resulted in differences between irradiated and un-irradiated samples at doses as low as 10 μM (Figure 1A). All three wt p53 HPV-negative cell lines responded in a similar fashion to PM2 therapy, with UM-SCC-74B demonstrating the most pronounced sensitivity with and without radiation. As expected, the two p53 mutated cell lines did not respond to PM2 monotherapy. Interestingly, even though A431 (which has a single point mutation of *TP53*) was unaffected by PM2 therapy alone, a significant decrease in viability was detected when combined with ionizing radiation (Supplementary Figure 1B). Some effect of Nutlin-1 on mutant p53 cells, albeit at considerably higher concentrations than required for wt p53 cell lines, has previously been shown in an *in vitro* study (37). Thus, it is possible that there is potential for combined PM2 and radiotherapy also for selected mutant p53 cancers. No response was observed on the HPV positive cell line UT-SCC-45 regardless of the addition of ionizing radiation. This is in line with previous studies indicating that the HPV E6 protein binds to p53, resulting in ubiquitin-dependent degradation and rendering MDM2-p53 inhibitors useless (38). In this study, only one HPV-positive cell line was investigated, and further studies are needed to confirm this finding. Furthermore, the lack of response when using the scrambled PM2 peptide (PM2^SCRAM^) validates the specificity of PM2 to its target (Supplementary Figure 1A). Taken together, these results support and validate previous findings on the specific MDM2/X-p53 antagonistic properties of PM2 (25, 27, 28).

Two of the main cellular responses following p53 activation are cell cycle arrest and apoptosis, the latter through transcription of pro-apoptotic proteins such as Noxa (14, 17, 37). In the present study, combination therapy (EBRT and PM2) prolonged and increased the amount of viable cells in the radiosensitive G2/M-phase and resulted in S-phase depletion (Table 2; Figure 1B). The prolonged arrest in G2/M-phase and S-phase depletion suggests that the irradiated cells are unable to repair the DNA damage and are more likely to undergo cell death rather than proceed through mitosis, further preventing cell proliferation (39, 40). These results demonstrate that the combination therapy of PM2 and EBRT resulted in greater effects and potency than either treatment alone.

Western Blot results confirmed PM2 specificity through an extensive increase in p53-levels following PM2 therapy. The upregulation of p53-expression is in concordance with our previous study (28) as well as studies using Nutlin-3, where increased p53-expression was directly correlated to increasing concentrations of the MDM2-p53 inhibitor (37). Combination therapy further increased wt p53 levels in both UM-SCC-74B and HCT116 samples (Figures 2A,D). UM-SCC-74B samples presented a near 5-fold increase in wt p53 levels following combination therapy. The extensive increase could be the reason why UM-SCC-74B was more sensitive both to PM2 therapy and to the combination treatment compared to HCT116 throughout the study. Four-fold and near-three-fold increases in cleaved caspase-3 levels were detected in PM2-treated UM-SCC-74B and HCT116 samples, respectively, indicating elevated apoptotic activity (Figures 2B,D). Interestingly, elevated levels of senescence were also detected, through increased levels of GLB-1 (Figures 2C,D). The GLB-1 levels followed the same pattern as p53, cleaved caspase-3 and Noxa, meaning the greatest increase was observed for the combination treated samples. However, the increase was less pronounced and failed to reach significance in the HCT116 samples. These results warrant further investigation of the mechanisms behind the possible induction of p53-mediated senescence pathways following PM2 therapy.

Flow cytometric analyses confirmed the increased levels of cleaved caspase-3 in both cell lines (Figure 2E). Increased levels of Noxa were detected in both UM-SCC-74B and HCT116, where the levels following the combination of EBRT and PM2 therapy proved greater than EBRT or PM2-monotherapy (Figure 2F). Interestingly, in samples treated with only EBRT, neither cleaved caspase-3 nor Noxa-expression levels differed from untreated controls, whereas PM2 therapy resulted in distinct increases at 72 h post-treatment. The increased levels of Noxa-expression of the PM2-treated samples suggest that the observed cell death could be the result of p53-mediated apoptosis. Thus, we conclude that the apoptotic effects induced by PM2 therapy are further potentiated by a combination with EBRT in wt p53 cancer cell lines. Moreover, the present study is the first to demonstrate increased senescence and p53-mediated apoptosis in cells treated with a combination of PM2 and EBRT, and may provide an explanation to the synergistic therapeutic effects obtained.

While monolayer assays are suitable for mechanistic evaluations, often generating greater therapeutic responses, anti-tumorigenic properties are better assessed in assays simulating the 3D-structure of tumors, such as *in vitro* 3D tumor spheroid models. When switching from monolayer to 3D models, the treatment schedule must be changed accordingly to better emulate an *in vivo* setting. As such, PM2-treatments were incubated for 48 h in all 3D assay settings to simulate the biological elimination and excretion rates *in vivo*. Treatment with PM2 every second day is less frequent than other MDM2-p53 inhibitors in current clinical trials (41). However, PM2 offers an extended biological half-life as well as inhibition of both p53 negative regulators MDM2 and MDMX. Therefore, less frequent doses of PM2 therapy could potentially circumvent issues of resistance and toxicities as seen with other MDM2-p53 inhibitors, for example Nutlin-3 (23, 26). Furthermore, treatment resistance can be avoided by combining PM2 therapy with EBRT.

In the present study, UM-SCC-74B spheroids irradiated with one, two, three or five times 2 Gy at 48 h intervals resulted in dose-dependent growth inhibition (Figure 3A). Interestingly, a single dose (20 μM) of PM2 incubated for 48 h, which in monolayer assays was highly potent in reducing viability, failed to impair UM-SCC-74B spheroid growth. This difference in PM2-potency is likely due to the changes in treatment schedule from monolayer to 3D setting (42). However, repeated doses at 48 h intervals did inhibit growth in a dose-dependent manner similar to that of repeated EBRT (Figure 3B). When combining the two therapies, potentiating effects were observed; a single 20 μM dose of PM2 (48 h incubation) which previously rendered little or no effect as a monotherapy, resulted in synergistic growth inhibitory effects for all treatments until day 14, with CDI < 0.7 when combined with either single or fractionated radiation treatments (Table 3; Figure 4). A further evolution of the combination regimens with both repeated EBRT and PM2 doses resulted in synergism in all combinations (Table 3). While all combinations resulted in growth inhibition, the majority of the spheroids eventually regained normal growth patterns (Figure 4). However, the rate at which they did differed markedly between the treatment regimes. There was a strong repeated-dose-dependent response to combination therapy: two repeated doses of each treatment resulted in growth inhibition surpassing the standard 14-day assay (data not shown), while adding a third dose of PM2 further increased the time to spheroid progression (Table 4; Figure 4). Three repeated doses of each therapy further prolonged the inhibitory effects, extending the median time to spheroid termination due to excess size to 28 days post-start of treatment, a 2-fold increase compared to untreated controls (Table 4). Most strikingly, five repeated doses of both therapies resulted in 100% spheroid disintegration (Figures 3C, 4). These findings demonstrate for the first time that, despite each treatment having an inhibitory effect alone, combining PM2 and EBRT can result in complete collapse of tumor spheroids and tumor cell death. This demonstrates the great potential of PM2 as a radiosensitizer in combination with fractionated EBRT, and may be especially suitable to increase the effect and reduce the frequency of recurrent tumors from small surviving subpopulations.

To conclude, the potential antitumorigenic effects of the combination of EBRT and PM2 therapy, as well as the mechanisms behind the effects and the cellular fates of treated cells were assessed in the present study. The study presents previously unknown data on PM2 and radiation treatment, demonstrating cell cycle arrest as well as upregulation of p53-mediated apoptosis. We therefore conclude that PM2 shows great promise as a future therapeutic cancer drug candidate, particularly as a radiosensitizer in combination with ionizing radiation.

## Data Availability Statement

All datasets generated for this study are included in the manuscript/Supplementary Files.

## Author Contributions

AM designed the study, acquired the majority of the data, performed data analyses, statistical analyses and interpretation and drafted the article. DS assisted with the design of the study, acquired data and performed analyses and interpretation, and revised the original draft. CB acquired data, performed data analyses and interpretation, and revised the original draft. DL assisted with the design of the study and performed interpretation of data as well as revised the original draft. MN designed the study with AM and performed data analyses and interpretation as well as revised the original draft. All authors have approved the submitted manuscript.

### Conflict of Interest

DL heads a collaboration with Merck on macrocyclic peptides. DL and CB have a patent EP3256484A1 pending. The authors declare no potential conflict of interest.

## Supplementary Material

Supplementary Figure 1 **(A)** Titrations of PM2 and PM2^SCRAM^ against HEK293 cells that are transiently transfected with the Mdm2:p53 NanoBIT system. Cells were treated for 4 h with either compound in 10% FCS containing DMEM cell media. IC_50_ of PM2 were determined to be 14.8 ± 0.5 μM using a 4-parameter model. Curve fitting was performed using Prism 8 (Graphpad). **(B)** Cell viability (XTT) response to treatment with 20 μM of PM2 of A431, H314, UT-SCC-45, and UM-SCC-74A cells with or without EBRT (2 Gy) *n* ≥ 4. Please note that irradiated cells were normalized to survival at 2 Gy, 0 μM PM2, whereas un-irradiated cells were normalized to survival at 0 Gy, 0 μM PM2 in order to compensate for the effects of radiation.
