# Supplementary figures and images for: The Stapled Peptide PM2 Stabilizes p53 Levels and Radiosensitizes Wild-Type p53 Cancer Cells
Source: Front Oncol. 2019 Sep 19;9:923. doi: 10.3389/fonc.2019.00923 (PMC6764291; doi:10.3389/fonc.2019.00923)

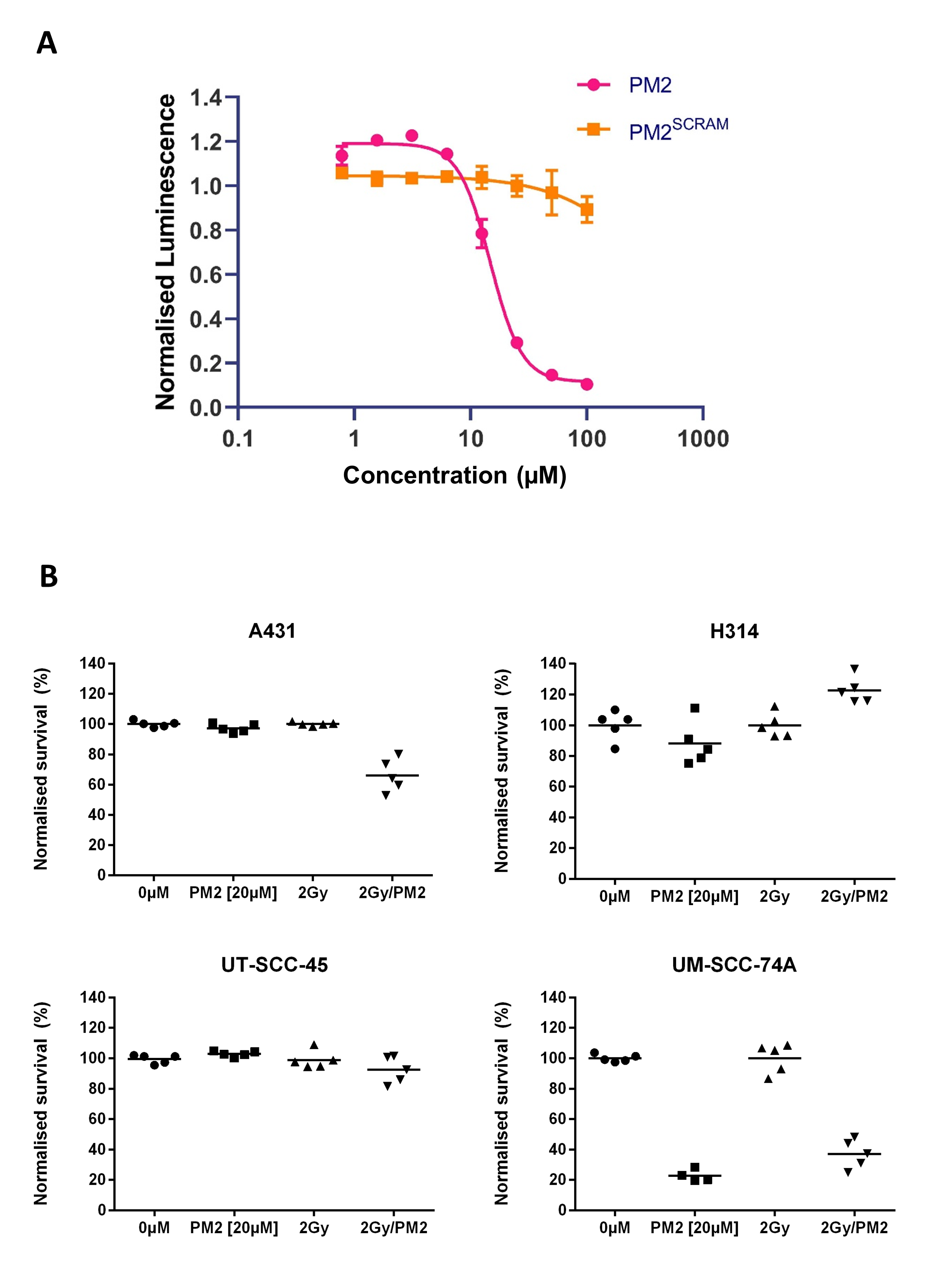

Supplement: Supplementary Figure 1 — (A) Titrations of PM2 and PM2SCRAM against HEK293 cells that are transiently transfected with the Mdm2:p53 NanoBIT system. Cells were treated for 4 h with either compound in 10% FCS containing DMEM cell media. IC50 of PM2 were determined to be 14.8 ± 0.5 μM using a 4-parameter model. Curve fitting was performed using Prism 8 (Graphpad). (B) Cell viability (XTT) response to treatment with 20 μM of PM2 of A431, H314, UT-SCC-45, and UM-SCC-74A cells with or without EBRT (2 Gy) n ≥ 4. Please note that irradiated cells were normalized to survival at 2 Gy, 0 μM PM2, whereas un-irradiated cells were normalized to survival at 0 Gy, 0 μM PM2 in order to compensate for the effects of radiation. [file Image_1.TIF]
